# Clinical effects of staged joint replacement in patients with septic arthritic knee

**DOI:** 10.1186/s13018-020-02062-1

**Published:** 2020-11-11

**Authors:** Ming Ni, Jun Fu, Tao Deng, Erlong Niu, Chi Xu, Xiang Li, Wei Chai, Guoqiang Zhang, Jiying Chen

**Affiliations:** The First Medical Centre, Chinese PLA General Hospital, Beijing, 100853 People’s Republic of China

**Keywords:** Knee, Septic arthritis, Spacer, Two-staged joint replacement

## Abstract

**Objective:**

To assess the clinical effect of staged joint replacement for the treatment of septic arthritic knee and the therapeutic differences between antibiotic cement beads and the tibial plateau spacer.

**Methods:**

Twenty-three patients (24 knee joints) treated with a staged joint replacement for septic arthritis knee were retrospectively reviewed between March 2014 and April 2018. At the first stage, thorough debridement and irrigation with self-made antibiotic cement beads or tibial plateau spacer were performed. After that, systemic antibiotic treatment was followed; when the infection was surely eliminated, the second-stage TKA was performed. Knee mobility (range of motion, abbreviated to ROM) and function (HSS scores system) were evaluated before surgery, in the interval period, and after joint replacement.

**Results:**

All patients finished follow-up, and the mean follow-up time was 27.3 months (12–54 months). Each group has one patient replaced with a homotypic spacer, and all patients eventually cleared the infection. None of the patients had a recurrent infection. The mobility and HSS scores of the two groups were significantly improved postoperation (*p* < 0.05). And there was no significant difference in the post-surgery ROM (*p* = 0.153) and the HSS score (*p* = 0.054) between the two groups.

**Conclusion:**

Staged joint replacement is an efficacious way for septic arthritic knees, whether tibial plateau spacer or antibiotic cement beads were used, which can effectively control infection and improve knee function.

## Introduction

Septic arthritic knee (SAK) is a relatively low-incidence disease while it has a high risk of disability and mortality [1]. There is currently no universally agreed ideal treatment strategy, and its treatment still faces great challenges [2]. Traditional treatment methods include knee incision or arthroscopic cleaning/irrigation/drainage based on systemic antibiotic treatment [3]. Even if anti-infective treatment against SAK is timely and appropriate, permanent joint destruction and persistent infections are still common [4]. For SAK cases with false infection control, rescue treatments, such as fusion and amputation, are often adopted [5, 6]. In recent years, there have been occasional reports of a method similar to the second-stage revision [7–10] for the treatment of SAK, including first-stage debridement and implantation of static or articulated spacer to control infection and second-stage initial total knee replacement to restore knee function [2, 5, 11–16].

Previous relevant studies are limited to case reports and small-sample size studies and lack the comparisons of spacer types. This study observed the effects of various spacers based on the same staged joint replacement surgery strategy, aiming to further demonstrate the effectiveness of staged joint replacement for the treatment of SAK and to explore and analyze the efficacy differences of different spacers.

## Materials and methods

### Patients

This study was approved by the ethics committee of the PLA General Hospital. Twenty-three SAK patients (24 joints) treated with staged joint replacement in the department of orthopedics in our hospital from March 2014 to April 2018 were retrospectively collected (Table 1). Nine patients (9 joints) were treated with antibiotic-containing tibial plateau spacer (group A), and 14 patients (15 joints) were treated only with antibiotic cement beads (group B). In group A, 1 patient had a history of open knee surgery, 5 patients had a history of knee injection, 1 patient had a history of arthroscopy, 2 patients had an unknown infection, and 8 patients had positive cultures (3 patients had a fungal infection). In group B, 3 patients had a history of trauma, 5 patients had a history of knee injection, 3 patients had a history of arthroscopy, 3 patients had unknown infection causes, and 6 patients had positive cultures (1 patient had a fungal infection, and 1 patient had a mixed infection).

**Table 1:** Basic information of the patients before surgery

Grouping (no.)	Gender/age (years)	Affected side	Preoperative HSS	Preoperative ROM	Reason for knee infection	Results of culture
A1	F/70	L	32	40	History of knee injection	*Candida albicans*
A2	F/60	L	48	100	History of knee injection	*Candida fris*
A3	F/69	L	35	40	History of knee injection	*Candida parapsilosis*
A4	F/65	L	41	20	Unknown	
A5	M/70	L	35	95	History of arthroscopy	*Micrococcus luteus*
A6	F/68	R	61	85	Unknown	*Propionibacterium acnes*
A7	M/58	L	36	61	History of knee injection	*Nocardia*, Gram-positive bacilli
A8	M/75	R	30	85	History of knee injection	*Staphylococcus warneri*
A9	M/45	L	14	70	History of knee incision for nail removal	*Staphylococcus surface*
Average of group A	4 M/5 F, 64.4	7L/2R	36.9	66.2		
B1	F/63	R	46	110	History of knee injection	
B2	F/66	R	28	50	Unknown	
B3	F/58	L	8	40	Unknown	*Staphylococcus aureus*
B4	F/49	R	46	90	History of arthroscopy	
B5 (two sides)	M/62	R	27	20	History of trauma	Gram-positive bacilli
		L	23	45		
B6	F/71	L	23	10	Unknown	*Staphylococcus aureus*
B7	F/71	L	32	40	History of knee injection	*Staphylococcus aureus*
B8	F/58	R	30	30	History of arthroscopy	*Aspergillus flavus*
B9	M/55	R	45	60	History of knee injection	
B10	F/57	R	20	20	History of knee injection	*Staphylococcus surface* and *Staphylococcus hominis*
B11	F/66	R	40	40	History of knee injection	
B12	F/48	L	35	60	History of knee injection	
B13	F/57	R	19	60	History of knee injection	
B14	F/55	R	35	40	History of knee injection	
Average of group B	2 M/12 F, 59.7	5L/10R	30.4	47.7		
Sum	6 M/17 F, 61.6	12 L/12R	32.9	54.6		

### Diagnostic criteria of SAK [15]

The diagnosis was combined with the individual medical history, together with the symptoms and signs of clinical infection (painful effusion, restricted mobility, elevated skin temperature, or the presence of the same sinus as the joint); elevated serum inflammation markers (C-reactive protein [CRP > 10 mg/dL], erythrocyte sedimentation rate [ESR] > 30 mm/h), polymorphonuclear (PMN) cell count percentage > 90%; imaging-revealed narrowing of joint space and destruction of articular cartilage; surgery-revealed purulent slip in the joint cavity, synovial membrane, or tissue; frozen sections (> 5 neutrophils/HPF) of suspicious infection during surgery; and positive results of synovial fluid or tissue culture.

### Inclusion criteria

The inclusion criteria are as follows: (1) the patient should be confirmed to have SAK; (2) anti-infection or other surgical methods were not effective; (3) the patient had obvious knee joint pain and limited joint movement. X-ray of the knee joint before surgery indicated KL ≥ 2 grade; (4) the patient had a preoperative evaluation of being able to tolerate surgery and had no mental illness; and (5) the patient fully understood the meaning and risks of staged surgery and signed relevant medical documents.

### Exclusion criteria

The exclusion criteria are as follows: (1) the patient had still good knee function and the symptoms were mild, (2) the patient had a complicated infection of the other parts (lung, urinary system, femur, tibia, etc.), and (3) the patient cannot complete staged joint replacement.

### Treatment

#### First-stage surgery

**Fig. 1:**
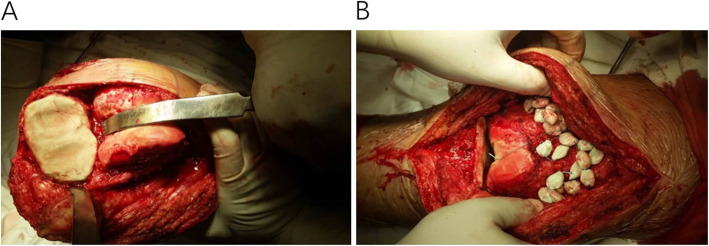
**a** Tibial plateau spacer implantation. **b** Antibiotic cement bead implantation

The median incision and lateral medial approach of the patella used in conventional TKA were performed. During the operation, 3 to 5 suspicious infected tissues in different parts were sampled for rapid intraoperative frozen slice examination; the results of which in all patients indicated that the knee joint was infected. Thorough debridement and repeated flushing with hydrogen peroxide, iodine, and saline were then performed within the reach of the surgical field. For the patients in group A (antibiotic methyl-methacrylate cement polymer, Heraeus Medical GmbH, Wehrheim/Ts., Germany), the tibial plateau was performed 9-mm osteotomy to fully expose and clean the joint capsule. A temporary prepared tibial plateau was fixed on the tibial plateau (Fig. 1a), and the antibiotic cement beads were placed on the front and sides of the knee capsule (Fig. 1b). The strategy for antibiotic use was the same in the two groups. For the patients with an unidentified pathogen, 4 g of vancomycin powder (VIANEX SA, Athens, Greece) + 2 g of meropenem (Sumitomo Dainippon Pharma Co. Ltd., Osaka, Japan) were placed in 40 g of bone cement. For the patients with known culture results before surgery, appropriate doses of antibiotics or antifungal drugs according to the results of drug sensitivity test were added. A negative pressure drainage tube was applied after the surgery was completed.

**Fig. 2:**
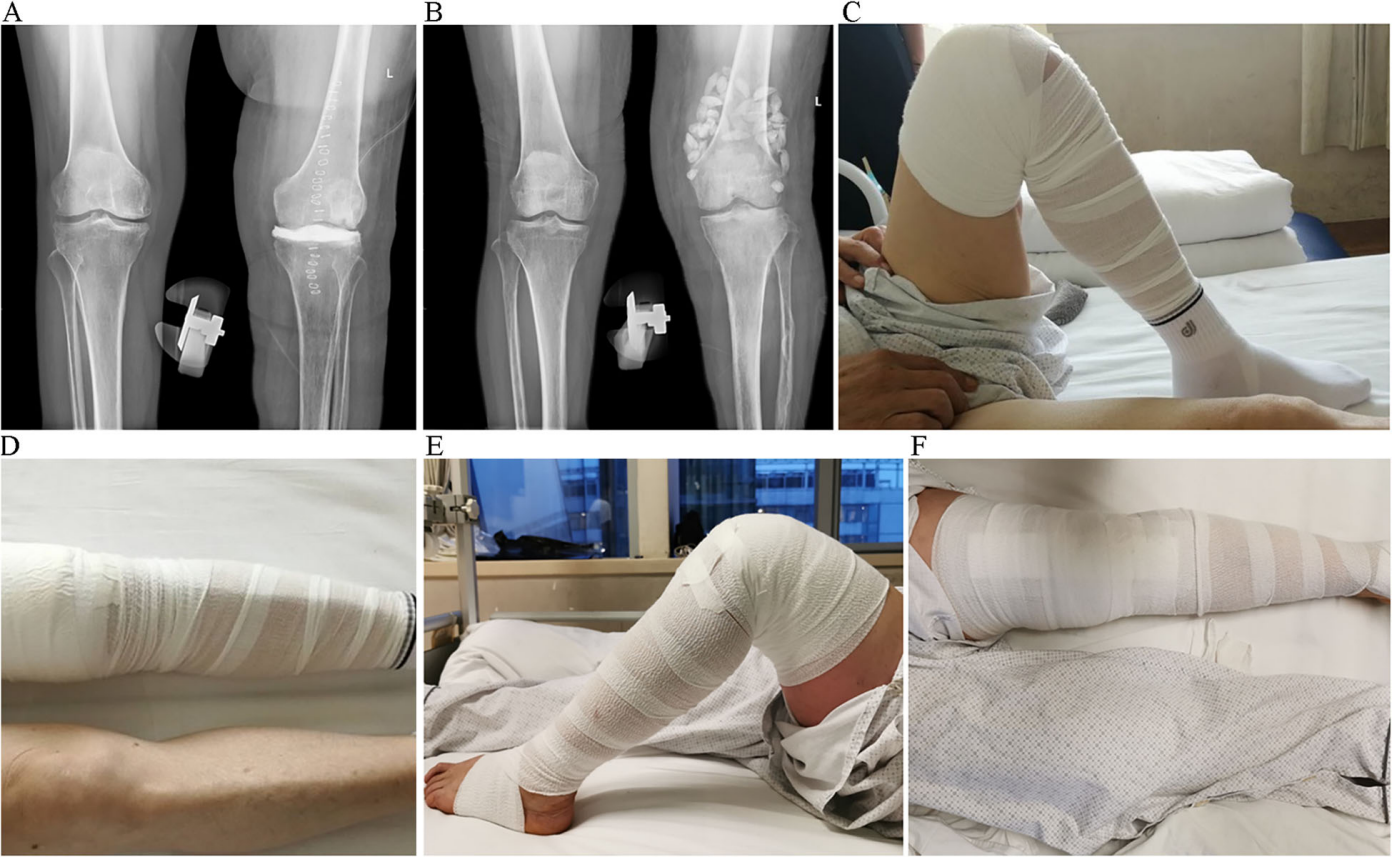
**a** X-ray of the knee joint after tibial plateau spacer implantation. **b** X-ray of the knee joint after antibiotic cement bead implantation. **c**, **d** Functional photos of a patient with tibial plateau spacer, whose ROM was improved from 10–100° preoperative to 0–110° during the interval period. **e**, **f** Functional photos of a patient with antibiotic cement beads, whose ROM was improved from 10–80° preoperative to 0–90° during the interval period

After surgery, the affected knee joint was kept straight, and the drainage tube was withdrawn when the drainage was less than 50 mL/day and clear. After drainage tube withdrawal, partial weight-bearing activities were allowed under the protection of knee braces. After surgery, 6-week routine intravenous broad-spectrum antibiotics (when the culture result was negative) or corresponding sensitive antibiotics were administrated; then, oral antibiotics were administrated for at least 6 weeks or until the clinical symptoms and signs disappeared and ESR/CRP returned to normal (Fig. 2). Once the clinical indexes were normal for two times, the spacer can be removed for knee joint replacement.

#### Second-stage surgery

The same original knee incision and original approach were used in the second-stage knee replacement surgery. After exposing the joints, 3 to 5 soft tissues of different parts were sampled for rapid intraoperative frozen slice examination. The results showed that the joint replacement can be performed as planned when the infection was ruled out. After removing the spacer, the joint cavity was fully washed and cleaned, and the surgery was then completed according to the method of the initial knee joint replacement. Because tibial plateau osteotomy had been completed in the patients with tibial plateau placer having been placed in first-stage surgery, this step can be omitted in the second stage. Postoperative treatment and functional exercise were performed in accordance with conventional TKA.

If the patient’s interval clinical evidence or second-stage intraoperative freezing slice test indicated persistent knee infection, the same bone cement spacer as before should be replaced again in combination with a systemic antibiotic for continuous anti-infective treatment. One patient in each group received such spacer replacement.

### Data collection

(1) The patients’ hospital medical history, relevant medical history, and relevant test results, as well as previous invasive knee operations, time, effects, and outcome, were reviewed; (2) the patients’ mobility and knee function scores (HSS scoring system) before and after surgery in the hospital were recorded; and (3) the patients were followed up after joint replacement, and the X-ray images of the knee joint, blood routine, ESR, CRP, joint mobility, and HSS score were reviewed.

### Statistical analysis

To evaluate the efficacy of staged surgery in each group, univariate analysis (ANOVA) was used to compare the differences in the range of motion (ROM) and functional score (HSS) before surgery, during the interval period, and after replacement. The SNK *t* test was then used to compare the functional and activity differences in each group between any two periods. In order to compare the differences in the efficacy between the two groups, the independent-sample *t* test was used to compare the function and ROM at the same period. All data results were analyzed using SPSS 20.0 with *p* < 0.05 being considered as statistical significance.

## Results

### General information

The detailed preoperative information is shown in Table 1. The average age was 64.4 ± 9.0 years in group A and 59.7 ± 7.2 in group B (*p* = 0.176). The body mass index was 26.1 ± 2.6 kg/m^2^ and 25.0 ± 5.6 kg/m^2^, respectively (*p* = 0.531). And the preoperative HSS and ROM were of no statistical significance in these two groups (Table 2).

**Table 2:** Comparison of ROM and HSS score between group A and group B during different stages

	ROM (°)			HSS		
	Preoperation	Interval period	Postoperation	Preoperation	Interval period	Postoperation
Group A	66.2 ± 27.9	57.8 ± 32.2	109.4 ± 18.1	36.9 ± 12.9	58.9 ± 11.4	90.5 ± 5.5
Group B	47.7 ± 26.2	69.7 ± 25.6	96.0 ± 23.3	30.5 ± 11.0	45.5 ± 14.0	80.9 ± 13.5
*p*	0.116	0.328	0.153	0.208	0.025*	0.054

*reflected *p*<0.05

All the patients were followed up for an average of 27.3 months (12–54 months). One patient in each group was re-debrided and replaced with a spacer due to persistent infection during the interval period, and after the infection was controlled, the second-stage knee replacement was completed. By the last follow-up, all the 23 patients (24 joints) had no recurrence of infection. The treatment success rate of staged replacement after a short-term follow-up was 100%.

### Comparison of knee HHS score and ROM during the interval period

The interval period was 6.7 months and 4.0 months in groups A and B, respectively (*p* = 0.073). In the interval period, the average HSS score of group A was significantly improved to 58.9 points (41–73 points), and the average HSS score of group B was also significantly improved to 45.5 points (23–73 points), which both showed statistical significance than those before surgery; what is more, the knee function score of group A was significantly higher than that of group B during the interval period (*p* = 0.025).

The average ROM of group A slightly decreased to 57.8° (20–110°), and the decrease was small and had no statistical significance. The average ROM of group B was significantly improved to 69.7° (10–100°). There was no statistical significance in ROM between group A and group B during the interval period (*p* = 0.328).

### Comparison of knee HHS score and joint mobility after second-stage TKA

At the last follow-up, the average HSS score of group A improved significantly to 90.5 points (83–95 points), and the average ROM increased to 109.4° (85–130°). The average HSS score of group B significantly improved to 80.9 points (63–95 points), and the average ROM increased to 96° (65–130°). There was no statistical significance in the HHS score (*p* = 0.054) and ROM (*p* = 0.153) between the two groups after the second-stage joint replacement. That is, the second-stage joint replacement surgery after infection control can restore the knee joint satisfactorily both in groups A and B, which has no statistical significance.

## Discussion

Treatment options for SAK are different from the type of infections. For acute knee infections, arthroscopic surgery is currently used [1]; for chronic joint infections or osteomyelitis, knee debridement is more suitable [5]; for periprosthetic joint infections, the generally accepted treatment is two-stage revision surgery [17–19]. However, for SAK, there is still no unified treatment strategy, and it is very challenging [20–22]. In response to this problem, a small number of studies have proposed staged joint replacement strategies similar to the two-stage revision. The treatment concept is the same as that of the two-stage revision. In the first stage, the infection is controlled by debridement and antibiotic cement spacer is implanted. After the infection is controlled, second-stage TKA surgery is performed to restore the knee joint.

Although research available for reference is limited, existing studies have shown that staged joint replacement can bring satisfactory infection control rate and knee function recovery against SAK, which is a promising treatment strategy for SAK. Nazarian et al. has reported 14 cases of non-articular spacer for staged joint replacement against chronic SAK with a success rate of 100% [5]. Shaikh et al. also reported 15 cases with SAK who all used joint-type spacer, among whom one case failed and was replaced with the same type of spacer, and another two cases refused to remove the spacer for the second-stage joint replacement due to satisfactory function after the infection was cured [14]. Yi et al. reported 17 cases of severe knee infection with a joint-type spacer, and only 1 failed [16]. Inadequately, previously reported staged operations have completed a large amount of osteotomy during the first stage of surgery, which is not conducive to the preservation of bone mass, and if the spacer is left in the body for a long period of time, there is a risk of its loosening or fragmentation, followed by the abrasion of bone mass. Studies have shown that long intervals can cause biofilm formation on the surface of the spacer and further cause continuous infection or reinfection [23–26]. Therefore, this study proposes the optimization with antibiotic cement beads and tibial plateau spacers.

A total of 23 patients (24 joints) were performed staged joint infection in this study. Nine patients (9 joints) were implanted with tibial plateau spacer, and 14 patients (15 joints) were implanted with antibiotic cement beads. After combined with systemic antibiotics, all the patients’ infection was effectively controlled. Compared with articulated spacer, tibial plateau spacer can achieve a thorough debridement while retaining more bone mass. The results show that whether using antibiotic cement beads or tibial plateau spacer as the local spacer, satisfactory knee infection control can be achieved, and the second-stage knee replacement surgery is especially for those with joint destruction or severe joint function impairment. However, for patients with SAK, the risk of directly performing joint replacement to form PJI is extremely high. The premise of staged knee replacement surgery must be the control of knee infection; otherwise, the failure rate of knee replacement will increase significantly. In this current study, the short-term follow-up showed the treatment success rate of staged replacement was 100%.

Theoretically, tibial plateau osteotomy has been performed during the implantation of the tibial plateau spacer, which can expose the joint cavity more fully and allow more thorough debridement of the posterior joint capsule, which is not possible by simply placing antibiotic cement beads. And the postoperative function in the tibial plateau spacer group was 90.5 points, which was 80.9 points in the antibiotic cement beads group. Due to the limited sample size, this study did not find a difference in infection clearance and postoperative function score between the two methods. However, both of them achieved satisfactory knee infection control and restored improved knee function.

There are still some limitations in this study. First, this retrospective study included a small number of cases and did not perform random grouping, which was limited by the low incidence of this disease and the lack of uniform treatment. However, we have included all our SAK patients eligible for second-stage surgery in the past 4 years. The total number of cases has exceeded the previous reports. This study included two types of spacer for subgroup comparison. Secondly, the duration of follow-up in this study varies, and certain patients did not reach more than 2 years of follow-up. The long-term efficacy still needs further follow-up; however, all the patients’ clinical manifestations, examination indicators, and intraoperative frozen slice test before replacement confirmed the control of infection, and there was no sign of recurrent infection at follow-up for at least 1 year. Therefore, the short-term treatment success rate was 100%.

## Conclusions

Treating SAK with staged joint replacement, whether tibial plateau spacer or antibiotic cement beads, can effectively control infection; joint replacement surgery after infection control can restore the knee joint satisfactorily. Therefore, we recommended staged joint replacement for SAK patients with symptomatic osteoarthritis.

## Data Availability

The datasets supporting the conclusions of this article are included within the article and its supplementary materials.

## Abbreviations

SAK: Septic arthritic knee
TKA: Total knee arthroplasty
HSS: Hospital for Special Surgery
ROM: Range of motion
PJI: Periprosthetic joint infection
